# Healthcare providers’ views on the acceptability of financial incentives for breastfeeding: a qualitative study

**DOI:** 10.1186/1471-2393-14-355

**Published:** 2014-10-09

**Authors:** Barbara Whelan, Kate J Thomas, Patrice Van Cleemput, Heather Whitford, Mark Strong, Mary J Renfrew, Elaine Scott, Clare Relton

**Affiliations:** Section of Public Health, School of Health and Related Research (ScHARR), University of Sheffield, Sheffield, S1 4DA UK; Medical Care Research Unit, ScHARR, University of Sheffield, Sheffield, S1 4DA UK; Mother and Infant Research Unit, College of Medicine, Dentistry and Nursing, University of Dundee, Dundee, DD1 4HJ UK

**Keywords:** Breastfeeding, Financial incentives, Qualitative, Acceptability, Relationships

## Abstract

**Background:**

Despite a gradual increase in breastfeeding rates, overall in the UK there are wide variations, with a trend towards breastfeeding rates at 6–8 weeks remaining below 40% in less affluent areas. While financial incentives have been used with varying success to encourage positive health related behaviour change, there is little research on their use in encouraging breastfeeding. In this paper, we report on healthcare providers’ views around whether using financial incentives in areas with low breastfeeding rates would be acceptable in principle. This research was part of a larger project looking at the development and feasibility testing of a financial incentive scheme for breastfeeding in preparation for a cluster randomised controlled trial.

**Methods:**

Fifty–three healthcare providers were interviewed about their views on financial incentives for breastfeeding. Participants were purposively sampled to include a wide range of experience and roles associated with supporting mothers with infant feeding. Semi-structured individual and group interviews were conducted. Data were analysed thematically drawing on the principles of Framework Analysis.

**Results:**

The key theme emerging from healthcare providers’ views on the acceptability of financial incentives for breastfeeding was their possible impact on ‘facilitating or impeding relationships’. Within this theme several additional aspects were discussed: the mother’s relationship with her healthcare provider and services, with her baby and her family, and with the wider community. In addition, a key priority for healthcare providers was that an incentive scheme should not impact negatively on their professional integrity and responsibility towards women.

**Conclusion:**

Healthcare providers believe that financial incentives could have both positive and negative impacts on a mother’s relationship with her family, baby and healthcare provider. When designing a financial incentive scheme we must take care to minimise the potential negative impacts that have been highlighted, while at the same time recognising the potential positive impacts for women in areas where breastfeeding rates are low.

## Background

In recent years rates of breastfeeding initiation in the UK overall have increased to over 80%. Despite this positive change, this increase is not reflected to the same extent in breastfeeding duration or exclusivity rates, with only 55% of women breastfeeding at six weeks and only 1% exclusively breastfeeding at six months [1]. This is despite the World Health Organisation recommending that women exclusively breastfeed their babies for six months and continue breastfeeding after the introduction of solid foods [2]. This recommendation is endorsed by the four UK Departments of Health [3–6]. There is a clear socioeconomic divide between those who breastfeed and those who do not. Mothers who are young, from low income backgrounds, of White ethnicity and with low educational attainment are least likely to breastfeed [1] due in part to them viewing breastfeeding as being embarrassing, inconvenient and not the cultural norm in the UK [7]. Rates of breastfeeding among these mothers have remained very low despite efforts to increase them, with the consequence that inequalities in health outcomes for these babies and their mothers are perpetuated [8].

Financial incentives have been used with varying degrees of success to encourage health-related behaviour change such as smoking cessation [9], smoking cessation in pregnancy [10], weight loss [11, 12] and attendance at health screening [13]. Research has found that incentives are particularly effective in encouraging people to take part in one-off activities such as preventive health checks, screening or immunisations, but the findings are less conclusive for more complex behaviours such as smoking cessation or weight loss [14]. The acceptability of financial incentives among the general public varies and is often dependent on the type of behaviour change that the incentive is targeting. Financial incentives for weight loss are more acceptable than incentives for smoking cessation [15]. Incentives for promoting healthy behaviour in pregnancy have been found to be more acceptable than for weight control for overweight people or illicit drug use reduction [16]. A recent study by Hoddinott et al. [17] explored people’s views around offering incentives to women who breastfed. They found that this was more acceptable to those of childbearing age, of non-White ethnicity and to those who had breastfed a previous baby.

There has been very little research on using financial incentives to encourage women to breastfeed. In Quebec, Canada women on benefits receive a monthly breastfeeding benefit of $55 (equivalent to approximately £30) until their baby is one year old [18]. Studies have examined the effect of giving gifts (approximate total value from $15-$100) to women who breastfed and attended breastfeeding educational programs in the United States [19, 20] or who had visits from breast feeding peer support workers in the UK (total value £71.99) [21]. These studies found positive results. Using financial incentives to increase rates of breastfeeding is different to using financial incentives for other health related behaviours in a number of ways. In the current context of the UK and other countries with low rates of breastfeeding, infant feeding is a complex and contentious behaviour. Studies have found that breastfeeding is strongly associated with a woman’s sense of identity as a mother [22], and involves a range of emotions such as dependency and trust between the mother and her baby [23]. In particular, a mother can experience feelings of guilt when breastfeeding does not go as planned or when she decides not to breastfeed [24, 25]. Murphy [26] has described infant feeding as a “moral minefield” with women being judged by others or themselves over their choice of infant feeding, young women have described it as ‘immoral’ to breastfeed in public [27], while Lee [28] has described the “breast is best” message as having a moralistic dimension in addition to its health message. Thus, adding a financial incentive into this environment can be seen as controversial and a “violation of a cultural norm” [29].

The findings, which are presented in this paper, are from qualitative research conducted during the intervention development stage of a wider study, planned to test the feasibility of offering financial incentives to encourage the initiation and continuation of breastfeeding in preparation for a cluster randomised controlled trial (ISRCTN44898617 – NOurishing Start for Health (NOSH)). The aim of the development stage, conducted between June 2012 and June 2013, was to inform the design of the financial incentive scheme by exploring the acceptability of such an intervention to key stakeholder groups, both in principle and in practice. As part of this development stage we interviewed healthcare providers with a range of infant feeding roles, either individually or in groups, and we present here the findings from these interviews. Other stakeholders were also interviewed, however, the findings from this will be reported elsewhere.

## Methods

### Design

This was a qualitative descriptive study, based on semi-structured individual and group interviews with health care providers involved in infant feeding. The aim of the study was to explore the acceptability in principle, and the perceived feasibility of, a hypothetical financial incentive scheme for breastfeeding. The aim of this paper is to describe healthcare providers’ views around the acceptability in principle of using financial incentives in areas with low breastfeeding rates. These healthcare providers were aware that they might be called on to implement a scheme as part of a cluster randomised controlled trial of financial incentives for breastfeeding.

### Setting

This study was conducted in Sheffield, South Yorkshire. The city had achieved full stage 3 (dual accreditation for hospital and community services) UNICEF UK Baby Friendly Initiative (BFI) accreditation a few months prior to the interviews being conducted [30].

### Participants

Participants were purposively sampled to include a wide range of experience and roles associated with supporting mothers with infant feeding. In particular, three strategies were used to purposefully select information rich cases [31]: politically important case sampling which allowed for key stakeholders who could be potential research collaborators to voice their opinions on the intervention; snowball sampling whereby interviewees identified key informants whose opinions were important to capture; and opportunistic sampling which allowed for the researchers to take opportunities during data collection to select potentially important cases. We consulted members of the local Maternal and Infant Nutrition group to ensure we included the full range of roles from within the statutory and voluntary sectors.

Information sheets were circulated to potential participants through gatekeepers in both the hospital and community setting. The gatekeepers included children’s centre managers, key members of staff in NHS Trusts and Local Authority breastfeeding co-ordinators. The researchers attended meetings of some professional groups in order to give information about the study and obtain the contact details of those who were present and interested in participating. Sampling of participants continued until no new perspectives on the acceptability of financial incentives for breastfeeding emerged.

### Data collection

Participants were invited by the researchers (BW and PVC) to either a group interview or an individual one-to-one interview. Participants sometimes preferred to take part in a group interview in order to be able to include other colleagues to get a wider breadth of views or because for convenience it was easier to interview a team of colleagues rather than each individually. Prior to interview all participants were asked to provide written consent after a further opportunity to have their questions answered. All participants agreed to have their interview recorded.

The interview topic guide contained the following items:

1. Opening questions about breastfeeding in general
  Infant feeding experience in current role
  Obstacles for women to starting and continuing breastfeeding
  Costs attached to breastfeeding

2. Opinion about financial incentives and the NOSH scheme
  Views on using financial incentives to promote health; and then for breastfeeding specifically?
  Incentive in form of cash or vouchers?
  How much and when (staged/lump sum)?
  Authorisation of cash/vouchers - verification criteria, who and how?
  Positives/negatives about the scheme?
  Any elements of the scheme of concern?
  Potential implications of the scheme: on the mother; family/close others; person responsible for giving the cash/vouchers?
  Additional factors to take into consideration?
  Support for such a scheme/what it would depend on?
  Impact of scheme on existing work and how?
  Likely acceptability to local population?

3. Any other comments

### Data analysis

All individual and group interviews were transcribed. Data analysis of all interviews was based on thematic analysis drawing on the principles of framework analysis [32]. Framework analysis is specifically designed to answer policy related questions and allows for rigorous and transparent data management. It is a structured process of thematic analysis that enables the identification of a priori and emergent themes which are grounded in the data, in that it is driven by the original accounts of the participants and the observations made by the researcher.

Two of the researchers (BW and PVC) read the first five transcripts and independently defined a preliminary thematic framework which they compared and reconciled where necessary. The initial framework was developed based on questions from the topic guide and the researchers’ observations and impressions. The framework was adapted accordingly as analysis proceeded. Each transcript was coded by the researchers (BW and PVC) in an iterative process. Themes and subthemes which emerged from the data were discussed in order to clarify and agree content and key overarching concepts were identified. In addition, preliminary findings were presented to a group of stakeholders for feedback as part of an information sharing event. The software package, NVivo 9, was used to enable data organisation and retrieval.

### Ethical approval

Ethics approval for the study was given by the School of Health and Related Research, University of Sheffield Research Ethics Committee (0591), by NHS Research and Development (SCH/12/078 and STH17193) and by the Sheffield Local Authority Research Governance Committee.

This study has adhered to the qualitative research review guidelines (RATS) [33].

## Results

In total, fifty-three healthcare workers, public health leads and commissioners from the NHS and Local Authority took part in either an individual interview (n = 37) or group interview (5 groups with 16 participants in total). This included midwives (including a student midwife) (n = 14); a nursery nurse (n = 1); breastfeeding peer support workers (including Local Authority and Action for Children employees) (n = 12); health visitors (including Family Nurse Partnership) (n = 7); children’s centre managers (including both Local Authority and Action for Children managers) (n = 5); NHS/Local Authority public health leads and commissioners (n = 5). Charity and voluntary sector workers were also interviewed (including National Childbirth Trust, doulas (in this context doulas support vulnerable expectant mothers throughout the last six weeks of their pregnancy, during the birth and six weeks postnatally) and health champions (volunteers who work to improve the general wellbeing of people living in the least healthy communities in the city) (n = 9).

The key theme emerging from healthcare providers’ views on the acceptability in principle of financial incentives for breastfeeding was their possible effect in ‘facilitating or impeding relationships’. There were several aspects to the overarching theme ‘financial incentives facilitating or impeding relationships’: the mother’s relationship with her healthcare provider and services, with her baby and her family, and with the wider community. For ease of presentation, the term ‘healthcare provider’ is used below to collectively describe healthcare workers, public health leads and commissioners from both the NHS and Local Authority. All quotes show the profession of the speaker in brackets and a participant number.

Before we present the findings on financial incentives facilitating or impeding relationships it is important to give some context with regards to the overall stance that healthcare providers took about financial incentives for breastfeeding. There were no stark differences between professional groups such as midwives and health visitors, but a similar range of disparate views was observed across individuals within each professional group. A minority of healthcare providers were either very positive or very negative about the scheme.

Of those who were very positive, a few referred to the success of financial incentive schemes to stop smoking in pregnancy and used this as an indication of the possible success of such a scheme to encourage breastfeeding. They were also convinced that it would work with certain target groups where breastfeeding rates remained low despite efforts to increase them. For those who were very negative, their main reason was not specific to breastfeeding, but instead they disagreed with giving financial incentives for health related behaviours that they felt people should do of their own volition.

*“I think people should do things for the sake of their health and the wellbeing of their children so I don’t like the idea of paying people to do what’s good for them to do anyway”* (NHS/Local Authority public health leads and commissioners, 37)

In addition, they could not see beyond possible difficulties with the practical implementation of the scheme, questioning whether the financial incentive might be spent on cigarettes and alcohol to “*feed another addiction*” or put undue pressure on women to breastfeed.

The majority of those interviewed described themselves as being *“on the fence” and* took a pragmatic view of the initiative. Many talked about the ethics of offering financial incentives for breastfeeding in terms of *“paying someone to do a behaviour that they should do anyway”* or possibly *“taking away people’s freedom of choice by putting that monetary value there”*. However, they also saw it as an opportunity to encourage women in areas where breastfeeding rates remain stubbornly low. Many recognised breastfeeding as a complex behaviour and wondered whether a financial incentive could possibly override all the other influences on breastfeeding such as *‘social, emotional, political, cultural and clinical elements’*. A few participants described being against the idea initially but having thought about it a little, subsequently changed their mind. Many felt that the incentive could help to increase the number of women who initiated and continued breastfeeding and this was viewed as being positive. In addition, during interviews, many indicated that they would be willing to be involved in implementing a scheme offering financial incentives to encourage breastfeeding in areas with low breastfeeding rates, as part of a piece of research.

*“I’ve been very much up and down about it … at first I was like no no, but the more I’ve thought about it the more I’ve thought, well I suppose the positive thing is people are going to breastfeed”* (Breastfeeding peer support worker, 4).

### Financial incentives for breastfeeding ‘facilitating or impeding relationships’

#### Mother’s relationship with her healthcare provider and services

Some viewed the financial incentive as a connector, either engaging the mother with breastfeeding support services or other services in the community.

*“It could only be a good thing, because whether they choose to do it or not, we’ve had that conversation with them about anything else that they might need from the Children’s Centre, or even encouraging them to access other kinds of support or groups or activities or whatever it is that they need at the point in time”* (Children’s centre manager, 9)

Others, particularly those involved directly in breastfeeding support, questioned whether it was *“right”* or *“ethical”* to give financial incentives for breastfeeding. Some spoke about this in general terms while others thought about the personal implications for themselves of offering a financial incentive to women. One healthcare provider involved in providing breastfeeding support likened the incentive to a *“hook”* which they could use to promote breastfeeding, but then questioned whether this was ethical. One midwife questioned whether they would be *“blackmailing”* women into breastfeeding by offering a financial incentive. A breastfeeding peer support worker discussed the incentive being like a bribe but also a possible reason for the woman engaging with her about breastfeeding.

*“It’d be like sort of, like a bribe kind of but I think they would want to talk to us more and it would be interesting to see if it made a difference and I think it would”* (Breastfeeding peer support worker, 18)

Most healthcare providers discussed verification of breastfeeding and how this could be done. Healthcare providers did not want to be responsible for *“policing”* the financial incentive scheme. Those in front line care, such as midwives and health visitors, raised concerns around how verification could jeopardise their relationship with a woman if they doubted her claims that she was breastfeeding. This was discussed particularly among health visitors who care for women from pregnancy through to when the baby is four years of age.

*“Having worked in areas with quite difficult families I could imagine finding that very challenging not wanting to ruin my relationship with the family to start saying I don’t believe you”* (Health visitor, 21)

Some health care providers discussed how breastfeeding peer support workers could play a role in verification as they have contact with women for a short time, so any negative impact would be short lived. Others saw it as an opportunity for women to engage with breastfeeding support services if the peer support worker was responsible for verification.

*“I suppose for me there’d be an expectation that that mum would engage with the breastfeeding peer support worker and that there would be some sort of relationship”* (Children’s Centre Manager, 6)

However, many breastfeeding peer support workers did not think that challenging women on whether they were breastfeeding was within their job remit and they discussed how they would hand this responsibility over to health visitors or midwives.

#### Mother’s relationship with her baby and her family

Healthcare providers also discussed how a financial incentive could impact on a mother’s relationship with her baby and her family. Some expressed concern about it having a negative impact on the mother-infant bond if it meant that women felt pressurised to breastfeed, particularly if their family was struggling financially.

*“If somebody really, really can’t stand to do it and it’s, you know, it’s affecting their relationship with the baby, you know, because every time the baby cried they resent it because they’ve got to put it to the breast and but they need that money or they really, really want to breastfeed but it’s just not working out”* (Children’s centre manager, 33)

In addition, they worried that if a woman had to stop (e.g. because they had to take medication contraindicated for breastfeeding), the incentive might exacerbate her feelings of guilt about stopping breastfeeding. One midwife painted a vivid picture of the incentive being like ‘*a noose that could be put around a mother’s neck*’, in the case where breastfeeding did not work out.

However, despite these concerns many healthcare providers felt that a financial incentive might help to increase the perceived value of breastfeeding, reinforcing other health promotion messages about the importance of breastfeeding. It may have a positive effect on family and friends in that they may encourage a woman to breastfeed.

*“If their family or their partner knew that they were going to get paid for it maybe they would encourage it”* (Health visitor, 19)

Some healthcare providers also discussed how it may help women justify breastfeeding, particularly if she was being pressurised to stop breastfeeding.

*“Maybe it would be like an extra defence, because I have seen mums who feel quite influenced by what their peers are saying, especially their mother, and maybe this will be an extra defence for them to keep going to with something that peer pressure around them has maybe, or the peer pressure might change, the peer pressure might be “oh you’re going to have to keep going a bit longer or else you’re not going to get your such and such vouchers””* (Midwife, 23)

But there were also fears that women may be coerced into breastfeeding by their partner or family. Some participants linked this with the issue of domestic violence where women who are already in abusive relationships may be further harassed to breastfeed because of the financial incentive.

*“Women may not always be the ones making the choice. There may be in a few cases coercion to do a particular type of thing whether it’s not to breastfeed or to breastfeed by a partners or families”* (Midwife, 16)

Because of these concerns many healthcare providers preferred the incentive to be vouchers rather than cash as vouchers would give the mother more ownership over the incentive. *“But if they’ve made their mind up that they really don’t want to I wouldn’t want then somebody in the family to be saying – you, you’ve got to because you’ll get an extra fiver … but I think there’d be less risk with vouchers”* (Children’s centre manager, 11)

#### Mother’s relationship with wider community

Many healthcare providers discussed how financial incentives could help make breastfeeding more normal and visible in communities where formula feeding was the norm. They felt that a mother receiving a financial incentive may be more inclined to discuss breastfeeding with friends and family.

*“On the plus side, the more mums that are breastfeeding the more socially acceptable it becomes, the more normal it becomes and so then it’s not going to be as hard a work to encourage people to breastfeed”* (Breastfeeding peer support worker, 2)

*“My personal view is it’s very positive because having worked in areas where breastfeeding isn’t part of the culture it might work”* (Health visitor, 21)

One charity/voluntary sector worker involved with breastfeeding support did not think that financial incentives would help normalise breastfeeding and was instead concerned that they would have the opposite effect making breastfeeding seem like something special that people did not usually do. A minority of participants, particularly those involved with breastfeeding at a strategic level within the city, worried that a financial incentive scheme would have a negative impact on existing work promoting breastfeeding. One midwife discussed how they were trying to encourage women to breastfeed through helping them *“feel the value of it from doing it and the response of their baby”* and she worried that a financial incentive would *“halt that process of culture change or alter its course”*.

## Discussion

Dykes and Flacking [34] have highlighted how important relationships are in encouraging breastfeeding, at the organisational, family, and staff-parent level. It is therefore not surprising that one of the key themes we identified in healthcare providers’ views of financial incentives for breastfeeding was around how financial incentives might facilitate or impede relationships. Thomson et al. [21] explored the effect of a breastfeeding incentive intervention in a disadvantaged area of North-West England and found that the incentive helped forge “connections” between women, families, peer supporters and health professionals. In our study, some healthcare providers viewed financial incentives as a potential ‘connector’ providing an opportunity for them to engage with women about breastfeeding or for women to engage with support services, particularly in children’s centres. This phenomenon of financial incentives leading to engagement with services has been observed before by Mantzari et al. [35] who found that women who were offered financial incentives to stop smoking in pregnancy engaged more with the Stop-Smoking Services than women not offered the incentives.

To date, research around the acceptability of financial incentives has mainly been conducted with members of the public [36, 15, 16] rather than specific groups such as healthcare providers. These studies have found that the public’s views of financial incentives for health related behaviour change are often polarised. In our study, a minority of healthcare providers expressed contradictory views. Some reflected on the success of financial incentives to encourage women to stop smoking in pregnancy and were convinced that they would work to encourage breastfeeding, while others could only identify the difficulties with implementing a financial incentive scheme and thought it wrong that people would be given money for something that they should do anyway. The majority of those interviewed remained ambivalent and took a pragmatic approach, identifying ways in which financial incentives could both facilitate and impede the mother’s relationships.

Our study brings a new perspective, that of the healthcare provider. Healthcare providers not only considered financial incentives from the perspective of the direct impact on the welfare of mothers and babies, but also from their professional viewpoint. This brought into question how incentives could impact on the professional integrity and responsibility of the healthcare provider towards women. Some feared that women would interpret an incentive as a form of bribery or blackmail and that this would impact negatively on their professional integrity and their relationship with the woman. In addition, they especially had concerns around verification and whether they would be responsible for “policing” a financial incentive scheme. A meta-synthesis of women’s perceptions and experiences of professional or peer breastfeeding support found that women identified an “authentic presence” as being the most effective support and that this was supported by building a trusting relationship with empathy [37]. Professional and peer support for breastfeeding are crucial in communities such as low income areas in the UK where breastfeeding is not the social norm [34]. It is unsurprising, therefore, that healthcare providers had concerns about how a financial incentive scheme would impact on their professional integrity and responsibility towards women. Verification of breastfeeding is quite unique in that other financial incentive schemes for smoking cessation [10] or weight loss [11] have an easily collected and objective measure of whether the target behaviour change is achieved. There is no such easily collected and objective measurement for breastfeeding and this was a concern voiced by interviewees. However, in the absence of such a measure, mothers’ self-report is currently used in infant feeding surveys, routine data collection and research. Breastfeeding can also be a sensitive issue and someone observing a mother breastfeed in order to verify that she is, could be regarded by the mother as being intrusive and insensitive. For these reasons observation of feeding by healthcare providers is unlikely to be accepted by either mothers or healthcare providers as ‘proof’ of breastfeeding.

Family and friends play an important role in a woman deciding whether to start or continue breastfeeding [38] and partners can be key in supporting women [39]. Healthcare providers in this study were concerned that a financial incentive scheme could mean that family members or partners might pressurise her to breastfeed in order to get the financial incentive and that this in turn could negatively impact on her relationship with her baby. While this was one view that was taken by some healthcare providers, many others saw the potential for the incentive to increase the value of breastfeeding among family and friends which could translate to them supporting a woman with breastfeeding. In a ‘stop smoking in pregnancy’ scheme in Scotland, Ballard and Radley [40] found that among women for whom smoking in pregnancy was a cultural norm, rewards to stop smoking in pregnancy gave mothers an excuse to opt out of the cultural norm within their peer group. A financial incentive for breastfeeding may have a similar effect.

Health promotion messages about the importance of breastfeeding are unlikely to motivate women to breastfeed if there is no acknowledgment of the “social, economic, psychological and cultural realities” [41]. Similarly, many healthcare providers thought it might not be enough just to offer a financial incentive, without acknowledging the wide range of factors which influence whether a woman breastfeeds or not. Some stakeholders involved in the promotion and support of breastfeeding were concerned that a financial incentive would impact negatively on local work which aims to protect, promote and support breastfeeding in line with UNICEF UK BFI standard [42]. Financial incentives for health related behaviour change cannot be used in isolation from other factors that influence behaviour change [43, 44] and should be used in combination with other supportive strategies such as patient education, training and social support [45]. Offering a financial incentive for breastfeeding could give a clear message about the value of breastfeeding for babies, mothers and society and the effort involved in breastfeeding and will need to work synergistically with other programmes in promoting and supporting breastfeeding.

### Strengths and limitations

This study has certain important strengths. It is the first study to explore healthcare providers’ views on financial incentives for breastfeeding. The sample included a large number of healthcare staff from a wide range of professional and organisational backgrounds and the qualitative aspect of the study provided the opportunity to explore their views on the acceptability in principle of using financial incentives for breastfeeding. There are also, however, limitations which include the (unavoidable) fact that the views expressed in the study are speculative rather than based on experience or knowledge. Once completed, the feasibility study will allow for a comparison to be made between healthcare providers’ views on a hypothetical scheme, as presented in this paper, and their views on a scheme that has been implemented in practice. This study includes the viewpoints of one stakeholder group, that of healthcare providers. The views of other relevant stakeholders such as women of childbearing age were sought during the development stage and these will be reported elsewhere.

## Conclusion

Relationships between mother, baby and family and also those between mother and healthcare providers lie at the heart of breastfeeding support theory and practice. It is therefore not surprising that the potential impact of financial incentives on such relationships emerged as a central theme of our analysis. A key priority for healthcare providers was that an incentive scheme would not impact negatively on their professional integrity and responsibility towards women. They believed that financial incentives could have both positive and negative impacts on a mother’s relationships with her family, baby and healthcare provider. When designing a financial incentive scheme we must take care to minimise the potential negative impacts that have been highlighted, while at the same time recognising the potential positive impacts. The majority of healthcare providers expressed a willingness to be involved in an experimental scheme which would measure the benefits of a financial incentive scheme for breastfeeding in areas with low breastfeeding rates. This indication of a pragmatic approach is borne out by the agreement of health visitors and midwives in South Yorkshire and North Derbyshire to participate in a feasibility study. The feasibility study is currently enrolling women to the experimental scheme and is due to report later in 2014.
